# Exploring the Role of Internalized Weight Bias as a Metric of Cumulative Social Disadvantage Among People With Obesity or Overweight: Insights From the OBSERVE Study

**DOI:** 10.1002/osp4.70082

**Published:** 2025-06-30

**Authors:** Jamy Ard, Julia P. Dunn, Hong Kan, Tracy J. Sims, Nadia N. Ahmad, Soohyun Hwang, Adam Jauregui, Sheila Drakeley, Scott Kahan, Neena A. Xavier

**Affiliations:** ^1^ Wake Forest University School of Medicine Winston‐Salem North Carolina USA; ^2^ Eli Lilly and Company Indianapolis Indiana USA; ^3^ Oracle Life Sciences Austin Texas USA; ^4^ George Washington University School of Medicine Washington District of Columbia USA

**Keywords:** body image, internalized weight bias, obesity, social determinants of health, weight self‐stigma questionnaire, weight stigma

## Abstract

**Background:**

Experiencing bias and stigma are social determinants of health (SDoH). Recently, internalized weight bias (IWB) has been included as an SDoH in obesity medicine. This study examined the association of IWB with other pillars of SDoH and how IWB contributes to cumulative social disadvantage for people living with obesity.

**Methods:**

A cross‐sectional, web‐based survey was conducted in the United States (May–December 2022) with a sample of adults with obesity or overweight. Multiple linear regression was performed to evaluate the relationship between WSSQ (higher score indicating experiencing more IWB) and other pillars of SDoH.

**Results:**

Participants who reported race as Black (adjusted mean difference [95% CI] −3.86 [−5.21, −2.52]; *p* < 0.001) or multiracial (−3.26 [−5.37, −1.16]; *p* = 0.002) had significantly lower total WSSQ scores than those who self‐identified as White race. Participants who experienced negative social interactions due to weight had significantly higher total WSSQ scores (7.04 [5.81, 8.27]; *p* < 0.001) than those who did not.

**Conclusion:**

The study showed that IWB is associated with certain other pillars of SDoH. Findings highlight the need for a comprehensive and patient‐centric approach to managing individuals with greater IWB, which may contribute to a higher cumulative social disadvantage and, consequently, worse health outcomes.

## 1 Introduction

Social determinants of health (SDoH) are defined by the World Health Organization (WHO) as “non‐medical factors that influence health outcomes. They are the conditions in which people are born, grow, work, live, and age, and the wider set of forces and systems shaping the conditions of daily life. These forces and systems include economic policies and systems, development agendas, social norms, social policies and political systems.” [1]. Decades of research have found that the burden of negative SDoH (i.e., higher SDoH index/score) is associated with worse health outcomes (i.e., morbidity, mortality, poor quality of life) [2, 3, 4, 5, 6, 7, 8]. Experiencing bias and stigma comprise one type of negative SDoH and are known to be key drivers of morbidity and mortality [9, 10, 11]. Experiencing bias and stigma can affect the sense of power and reinforce social stratification, thereby negatively impacting physical and mental health.

Weight stigma is defined as the discriminatory acts that stem from weight bias—negative beliefs about individuals due to their weight or size [12]. Experiencing such stigma across the lifespan can influence people to internalize negative beliefs, known as internalized weight bias (IWB) [13, 14]. A plethora of research has shown that greater IWB is associated with increased risks of depression and anxiety, poor body image [14, 15], higher hemoglobin A1c [16], increased C‐reactive protein [17], and poor maintenance of weight reduction [18]. Furthermore, there has been a demonstration that IWB mediates the relationship between experienced weight stigma and biopsychosocial outcomes [19].

The World Obesity Foundation (WOF) released a position statement indicating “weight‐related stigma is both an SDoH and human rights issue” [12]. Additionally, according to the American Association of Clinical Endocrinology consensus statement, IWB and weight stigma are both drivers and complications of obesity, and addressing IWB is necessary for optimal obesity care [20]. Experiencing bias and stigma are well‐documented components of SDoH that are widely recognized in both clinical practice and literature [9, 10, 20]. SDoH have also been shown to cluster, indicating that highly vulnerable patients experience worse outcomes based on non‐medical factors [4, 5, 6, 7, 8]. Additionally, cumulative social disadvantage, identified by high SDoH burden, predicts disease including increased risk of obesity [6]. Assessments for SDoH currently do not specifically evaluate for experienced weight bias and stigma [20, 21]. Therefore, the current analysis explored associations between IWB and other established pillars of SDoH to underscore the potential relevance of IWB to the social disadvantage burden of patients living with obesity, which remains underrecognized in clinical practice.

## 2 Methods

### 2.1 Data Source

This cross‐sectional, web‐based survey was conducted as a part of the United States (US) PerceptiOns, Barriers, and OpportunitieS for Anti‐obEsity Medications in Obesity CaRe: A SurVEy of Patients, Providers, and Employers (OBSERVE) study to assess perceptions of obesity, anti‐obesity medications (AOM), and experienced weight‐related bias and stigma [22, 23]. The survey results from participants living with obesity or overweight were included in the current analysis. Survey questions were informed by learnings from in‐depth qualitative interviews [24]. The survey data were collected from May 2022 to December 2022.

The study participants were recruited from an online recruitment platform (Kantar Profiles' survey panel, LifePoints). Potential participants were contacted via email and invited to participate in a 45‐min web‐based survey. The survey was available in both English and Spanish. Participants were assessed for eligibility through a screening questionnaire; eligible participants provided their informed consent electronically and proceeded to the main survey. All participants completing the survey were compensated for their time with a fair market value incentive. The study was granted exempt status from the Sterling Institutional Review Board (ID#9434) in November 2021. For this analysis, a subset of the overall survey questions, including questions from the WSSQ, were used.

### 2.2 Study Sample

The study included US adults (aged ≥ 18 years) either overweight (body mass index [BMI] 27 to < 30 kg/m^2^) having one or more obesity‐related complications (e.g., type 2 diabetes, cardiovascular disease, or hyperlipidemia) or with obesity (BMI ≥ 30 kg/m^2^). For Asian participants, overweight was defined as a BMI of 23.0–27.4 kg/m^2^ and obesity as BMI ≥ 27.5 kg/m^2^ [25]. Quotas (age, sex, race, ethnicity, and region) were set to represent a broad sample of US adults with overweight and obesity [26]. Individuals employed as clinicians were excluded from the study.

### 2.3 Measures

#### 2.3.1 Internalized Weight Bias: Weight Self‐Stigma Questionnaire Score

The WSSQ total score was used to assess IWB in participants with obesity or overweight [27, 28]. The WSSQ is a 12‐item scale that consists of Likert scale questions (rated 1 = completely disagree to 5 = completely agree) focusing on feelings related to shame or guilt about one's body. It consists of two subscales, each with 6 items, for measuring weight‐related self‐devaluation (item numbers 1–6) and fear of enacted stigma (item numbers 7–12). The total score, which is reported in the results, is calculated as the sum of all the items (1 through 12) and ranges from 12 to 60. Higher scores indicate experiencing more stigma related to one's perception of their body or weight [27, 28]. The WSSQ total score demonstrated sufficient internal reliability, with a Cronbach's *α* of 0.878 [28]. The scores for the two WSSQ subscales are provided in Table S2.

#### 2.3.2 Social Determinants of Health

Survey questions from the larger OBSERVE study were retrospectively mapped to six established SDoH pillars using two comprehensive frameworks: the Kaiser Family Foundation and WHO [29]. These two frameworks were selected because they provide a structured and widely recognized approach to understanding the various social, economic, and environmental factors that influence health outcomes. The mapping process involved reviewing and categorizing survey questions based on their relevance to established SDoH pillars, as outlined in Table S1. Socioeconomic position was assessed using data on gender, race/ethnicity, education level, and household income, while economic stability was evaluated based on whether the costs of certain weight management modalities impact long‐term success. Community and social interactions were examined through questions on social interactions, support systems, and experiences with healthcare providers and staff. Consideration of neighborhood and the physical environment focused on access to spaces for exercise, whereas the food environment was assessed based on the cost of healthy food and whether limited options affect weight management. Lastly, health and system were assessed using data on the quality of obesity care.

#### 2.3.3 Health Characteristics

Other health‐related characteristics that were self‐reported in the survey included prior AOM experience, prior weight management medical procedures (e.g., gastric bypass surgery, gastric band, etc.), weight and height, and participants' self‐reported obesity‐related complications (e.g., depression and anxiety).

### 2.4 Statistical Analyses

All study variables were summarized using descriptive statistics. Categorical data were reported as frequencies and percentages, and continuous variables were reported as means with standard deviations (SDs). Multiple linear regression was performed to evaluate the relationship between WSSQ and the pillars of SDoH while controlling for other relevant covariates. The covariates were selected based on their potential influence on IWB and interactions with SDoH as identified by empirical evaluation. Specifically, we conducted bivariate analyses to assess potential covariates' associations with IWB and SDoH, ensuring that the included covariates were statistically relevant to WSSQ. These covariates provide a comprehensive adjustment for individual health variations, ensuring a more precise examination of the association between IWB and other SDoH. Data were represented as adjusted mean differences, standard errors (SE), and 95% confidence intervals (CI). A *p*‐value of < 0.05, two‐tailed, was considered to be statistically significant. All analyses were conducted using R v4.2.0 or higher (R Foundation for Statistical Computing, Vienna, Austria).

## 3 Results

### 3.1 Participant Characteristics

Of the 1007 participants with obesity, 977 were included in this analysis. Excluded individuals were those who responded “prefer not to answer” to some of the demographic questions (e.g., gender, education, household income) to ensure the coherence and comparability of the categories within our covariates. The majority of study participants were female (64.1%), and less than half were White (41.4%) (Table 1). At the time of the survey, 69.2% reported having less than a BA/BS degree, and 43.1% had an income of $30,000–$75,000. The distribution of participants' BMI categories was overweight (15.7%), obesity class I (39.3%), class II (22.2%), and class III (22.8%). A total of 36.7% of participants reported an obesity‐related complication count of ≥ 4, and 28.9% reported having both depression and anxiety.

**TABLE 1:** Sociodemographic and health characteristics of the participants.

Characteristics	Overall (*N* = 977)
Age, years, *n* (%)	
18–39	354 (36.2)
40–64	467 (47.8)
65+	156 (16.0)
Gender, *n* (%)	
Male	351 (35.9)
Female	626 (64.1)
Race/Ethnicity, *n* (%)	
White	404 (41.4)
Black	286 (29.3)
Asian	65 (6.7)
Hispanic	147 (15.0)
Multiracial/Other/PNA	75 (7.7)
Number of obesity‐related complications, *n* (%)	
0	138 (14.1)
1	163 (16.7)
2	177 (18.1)
3	140 (14.3)
4+	359 (36.7)
Education, *n* (%)	
Less than BA/BS degree (some college or trade school, or AA/AS degree or trade school grad. or less)	676 (69.2)
BA/BS degree or above	301 (30.8)
Income, *n* (%)	
< $30k	326 (33.4)
$30k to ≤ $75k	421 (43.1)
> $75k	230 (23.5)
Baseline BMI, *n* (%)	
Overweight (≥ 27.0–29.9)	153 (15.7)
Obesity class I (≥ 30.0–34.9)	384 (39.3)
Obesity class II (≥ 35.0–39.9)	217 (22.2)
Obesity class III (≥ 40)	223 (22.8)
Self‐reported clinician diagnosis of depression/anxiety	
Neither	496 (50.8)
Depression only	72 (7.4)
Anxiety only	127 (13.0)
Depression and anxiety	282 (28.9)

Abbreviations: AA/AS, associate degree; BA/BS, bachelor's degree; BMI, body mass index; PNA, prefer not to answer; SD, standard deviation; TS, trade school.

### 3.2 Socioeconomic Position (e.g., Gender, Race/Ethnicity)/Economic Stability (e.g., Expenses, Employment) and Total WSSQ Score

Participants who self‐identified as Black race (adjusted mean difference [95% CI] −3.86 [−5.21, −2.52]; *p* < 0.001) or multiracial (−3.26 [−5.37, −1.16]; *p* = 0.002) had significantly lower total WSSQ scores than those who self‐identified as White race.

Participants who agreed that the costs of bariatric surgery affect weight management had significantly higher total WSSQ scores than those who disagreed (1.76 [0.40, 3.11]; *p* = 0.011) (Table 2).

**TABLE 2:** Multivariable regression analysis for WSSQ total score and SDoH covariates.

SDoH pillar	Characteristics	*N*	Total WSSQ core		
			Adjusted mean differences (SE)	95% CI	*p*‐value
	(Intercept)	977	25.57 (1.5)	22.71, 28.42	< 0.001
Socioeconomic position	Gender				
	Male	351	—	—	
	Female	626	0.10 (0.6)	−1.07, 1.27	0.870
	Race/Ethnicity				
	White	404	—	—	
	Black	286	−3.86 (0.7)	−5.21, −2.52	< 0.001
	Asian	65	−0.60 (1.2)	−2.92, 1.72	0.610
	Hispanic	147	−1.51 (0.9)	−3.17, 0.15	0.075
	Multiracial/Other/PNA	75	−3.26 (1.1)	−5.37, −1.16	0.002
	Highest education completed				
	Some college or TS, or AA/AS degree or TS grad. or less	676	—	—	
	BA/BS degree or above	301	1.13 (0.7)	−0.16, 2.41	0.085
	Household income level				
	> $75k	230	—	—	
	< $30k	326	0.26 (0.8)	−1.33, 1.84	0.750
	$30k to ≤ $75k	421	−1.01 (0.7)	−2.44, 0.42	0.170
Economic stability	Costs of WL meds affect weight management^a^				
	No	573	—	—	
	Yes	404	0.02 (0.7)	−1.42, 1.46	0.980
	Costs of lifestyle changes affect weight management^a^				
	No	381	—	—	
	Yes	596	0.11 (0.7)	−1.29, 1.52	0.870
	Costs of bariatric surgery affect weight management^a^				
	No	595	—	—	
	Yes	382	1.76 (0.7)	0.40, 3.11	0.011
Community and social interactions	Experiencing problems with social interactions due to weight^b^				
	Disagree	623	—	—	
	Agree	354	7.04 (0.6)	5.81, 8.27	< 0.001
	Nobody to participate with for weight management journey^b^				
	Disagree	508	—	—	
	Agree	469	2.62 (0.6)	1.39, 3.86	< 0.001
	Negative treatment by clinician/staff due to weight^b^				
	Disagree	813	—	—	
	Agree	164	2.92 (0.8)	1.41, 4.43	< 0.001
Neighborhood and physical environment	Lack of access to exercise affects weight management^c^				
	No	546	—	—	
	Yes	431	1.79 (0.7)	0.47, 3.10	0.008
Food environment	Healthy food being too expensive affects weight management^c^				
	No	338	—	—	
	Yes	639	0.54 (0.7)	−0.79, 1.87	0.430
	Limited healthy food options in neighborhood for weight management^c^				
	No	560	—	—	
	Yes	417	1.48 (0.7)	0.17, 2.78	0.026
Health and system	Clinician discusses weight management with patient				
	Sets WL goals that feel achievable	226	—	—	
	Does not discuss	293	−0.15 (0.8)	−1.67, 1.38	0.850
	Sets WL goals that feel unachievable/discusses weight management but doesn't set WL goals	458	0.74 (0.7)	−0.66, 2.14	0.300
Other control variable	Prior weight management medical procedure				
	No/PNA	895	—	—	
	Yes	82	−0.96 (1.0)	−2.95, 1.04	0.350
	Baseline BMI				
	Overweight (≥ 27.0–29.9)	153	—	—	
	Obesity class I (≥ 30.0–34.9)	384	0.92 (0.8)	−0.68, 2.53	0.260
	Obesity class II (≥ 35.0–39.9)	217	1.68 (0.9)	−0.12, 3.49	0.067
	Obesity class III (≥ 40)	223	3.15 (0.9)	1.33, 4.98	< 0.001
	Prior AOM experience				
	Never/Don't remember	779	—	—	
	Have taken/Currently taking	198	1.50 (0.7)	0.10, 2.91	0.036
Other control variable	Number of obesity‐related complications				
	0	138	—	—	
	1	163	0.75 (1.0)	−1.24, 2.73	0.460
	2	177	−0.06 (1.1)	−2.16, 2.03	0.950
	3	140	0.42 (1.1)	−1.81, 2.65	0.710
	4+	359	1.22 (1.1)	−0.96, 3.39	0.270
	Depression/Anxiety				
	Neither	496	—	—	
	Depression only	72	−0.92 (1.1)	−3.11, 1.28	0.410
	Anxiety only	127	1.62 (0.9)	−0.19, 3.43	0.080
	Depression and anxiety	282	1.29 (0.8)	−0.36, 2.94	0.130
	Age by group				
	40–64	467	—	—	
	18–39	354	0.58 (0.7)	−0.73, 1.88	0.390
	65+	156	−0.61 (0.8)	−2.26, 1.05	0.470

*Note:* “Weight management” refers to “keeping weight off over the long‐term (at least 2 years).”

Abbreviations: AA/AS, associate degree; AOM, anti‐obesity medications; BA/BS, bachelor's degree; BMI, body mass index; CI, confidence interval; PNA, prefer not to answer; SDoH, social determinants of health; SE, standard error; WL, weight loss; WSSQ, weight self‐stigma questionnaire; TS, trade school.

^a^ “No” was collapsed from response options “Not at all,” “A little bit,” and “I'm not sure/Does not apply”; “Yes” was collapsed from response options “Moderately,” “Quite a bit,” and “Very much.”

^b^ “Disagree” was collapsed from “Strongly disagree,” “Disagree,” and “Neither agree nor disagree”; “Agree” was collapsed from response options “Agree” and “Strongly agree.”

^c^ “No” was collapsed from response options “Not at all” and “A little bit”; “Yes” was collapsed from response options “Moderately,” “Quite a bit,” and “Very much.”

### 3.3 Community and Social Interactions (e.g., Support Systems, Community Engagement) and Total WSSQ Score

Participants who agreed that they experienced problems with social interactions due to their weight had significantly higher total WSSQ scores (7.04 [5.81, 8.27]; *p* < 0.001) than those who disagreed. Similarly, those who agreed that there was nobody to participate with in their weight management journey (2.62 [1.39, 3.86]) and felt that they had been treated negatively by healthcare providers/staff due to weight (2.92 [1.41, 4.43]) had higher total WSSQ scores than those who disagreed (both *p* < 0.001).

### 3.4 Neighborhood and Physical Environment (e.g., Walkability, Parks, and Playground)/Food Environment (e.g., Hunger, Access to Healthy Options) and Total WSSQ Score

Participants who agreed that lack of access to exercise in the neighborhood affected their weight management had higher total WSSQ scores (1.79 [0.47, 3.10]; *p* = 0.008) than those who disagreed.

Participants who agreed that limited healthy food choices in the neighborhood affected their weight management had higher total WSSQ scores (1.48 [0.17, 2.78]; *p* = 0.026) than those who disagreed.

### 3.5 Additional Findings and Total WSSQ Score

Participants in the BMI category of obesity class III had higher total WSSQ scores than those in the overweight category (3.15 [1.33, 4.98]; *p* < 0.001), while no significant relationship was observed for obesity classes I and II when compared to the overweight category. Also, participants who had been previously treated with or were being treated with an AOM at the time of data collection had higher total WSSQ scores (1.50 [0.10, 2.91]; *p* = 0.036) compared to those not on any medication or did not remember taking them.

## 4 Discussion

The current study demonstrated that certain established pillars of SDoH, including community and social interactions, socioeconomic position/economic stability, and neighborhood and physical environment/food environment, were associated with IWB. For community and social interactions, participants experiencing problems with social interactions due to their weight and those having no support during their weight management journey had significantly higher total WSSQ scores, indicating a higher degree of IWB. This is consistent with previous research, which has reported inequitable treatment and lack of support due to weight stigma in the workplace, healthcare settings, and interpersonal relationships with family and friends [13, 14]. These repeated negative social interactions could cause a social stratification for individuals living with obesity and high IWB through differential exposure and/or differential vulnerability to the damaging consequences of obesity. Overall, this work highlights that among PwO, the burden of experienced weight stigma and bias contributes to their cumulative social disadvantage, reflected in total SDoH burden. The significant associations of IWB and certain other SDoH pillars could inform how to identify and assist patients with increased IWB, particularly given the current lack of evidence on how to optimally address IWB [20].

In the same vein, participants who agreed that they faced negative treatment by clinicians/healthcare staff due to weight had a significantly higher total WSSQ score. These results are in line with prior studies in which adults with obesity were found to face stigmatization by healthcare personnel [30]. It has been reported previously that 42% of individuals with obesity were uncomfortable discussing their weight with clinicians, and only 26% reported that they were treated with respect when they sought advice from clinicians regarding their weight [13]. The potential distress from interactions with healthcare personnel within the community and social interactions pillar of SDoH can discourage patients from seeking care to manage obesity, influence trust between clinicians and patients [31], and further exacerbate vulnerability to health‐related complications.

While certain SDoH were positively associated with IWB, negative associations were also identified. Within socioeconomic position, Black and multiracial participants had significantly lower total WSSQ scores, indicating lower IWB than their white counterparts. While this may suggest a protective effect against internalized stigma, it does not necessarily mean that Black individuals experience less external weight stigma or fewer obesity‐related health inequities. The lower IWB among Black and multiracial participants observed in this study may reflect cultural differences in body image norms and weight perception, as research has shown that some Black communities tend to have more accepting or flexible attitudes toward larger body sizes compared to White populations [13, 32, 33]. Previous research also suggested that Black individuals with obesity experience similar weight stigma as their White counterparts but have less IWB and different approaches to coping [34]. Future research should explore how these cultural and systemic factors influence IWB and obesity treatment engagement, guiding more effective and culturally sensitive interventions [35, 36].

Thirty‐nine percent of participants reported that the cost of bariatric surgery affected their weight management, and this was associated with increased IWB. This relatively high proportion of participants who had considered bariatric surgery—despite high IWB—and had inquired about the costs is surprising, given that < 1% of eligible adults with obesity undergo bariatric surgery. In general, misperceptions about bariatric surgery [37] remain prevalent, including participants in the overall OBSERVE study [23]. Similarly, nearly 20% of participants reported prior AOM use, which was also associated with increased IWB. The directionality of the association between the cost of bariatric surgery and high IWB is unclear from this retrospective analysis. However, the fact that some individuals with high IWB are interested in pursuing therapeutic interventions—either through prior AOM use or questioning the cost of bariatric surgery—suggests the need for further research to understand how to best engage and support this subpopulation of patients.

Interestingly, some of the more traditional SDoH, such as income, education, and cost‐related barriers (e.g., cost of weight loss medications, lifestyle changes, and healthy food), other than the costs of bariatric surgery, were not significantly associated with IWB in this study. This may suggest that while financial and educational barriers are critical to overall health and weight management, PwO experience internalization of weight stigma regardless of social standing. Prior research has shown that experiences of weight stigma—particularly in healthcare settings, workplaces, and personal relationships—are powerful drivers of IWB, independent of socioeconomic position [14, 38]. Given these findings, it is important to consider that stigma itself represents a structural determinant of health, shaping access to quality healthcare, social support, and self‐perception in ways that contribute to health inequities [12]. Further research exploring the relationship between IWB and other pillars of SDoH is needed.

Greater SDoH burden is associated with detrimental physical and mental health outcomes, which has prompted healthcare systems and clinicians to increasingly identify and address SDoH as part of comprehensive patient management. The WOF's position statement, recognizing weight bias and stigma as SDoH, is an important call to action. With the knowledge that IWB is a mediator between experiencing weight stigma and bias and adverse health outcomes, it provides an opportunity to better define the cumulative burden of social disadvantage experienced by PwO. Our findings suggest that certain SDoH pillars such as community and social interactions, socioeconomic position/economic stability (i.e., race/ethnicity, bariatric surgery costs), and neighborhood and physical environment/food environment (i.e., lack of access to exercise, limited healthy food options) are associated with a higher degree of IWB. Further assessment, possibly leveraging the WSSQ, may provide additional evidence of IWB. Identifying and addressing modifiable components of SDoH would guide clinicians toward holistic obesity management, fostering a more intentional long‐term treatment plan and goals that ultimately improve health outcomes.

The main strength of this study was that it included a large sample size with diverse participants, including offering the option to complete the survey in Spanish. This study utilized a quantitative research design with learnings from qualitative interviews to inform the survey questions. However, the study was limited by the cross‐sectional design, and the sample was limited to online participants who may have different experiences and perspectives, as well as varying exposure to weight‐related stigma. Additionally, the OBSERVE study was not explicitly designed to identify the six SDoH pillars of interest, which may limit the comprehensiveness of the findings related to these domains. Additionally, the survey content addressing some of the SDoH pillars may not have been robust, potentially impacting the depth of insights gained from the study. Future research should consider a more targeted approach to ensure a comprehensive assessment of all relevant SDoH. Lastly, extensive clinical trial data on AOMs have been released since data collection, which may have impacted weight‐related stigma.

## 5 Conclusion

The present exploratory analysis from the OBSERVE study showed the potential role of applying IWB to measure cumulative social disadvantage in PwO. The study found associations between IWB and established pillars of SDoH, namely community and social interactions, socioeconomic position/economic stability, and neighborhood and physical environment/food environment in people with obesity. Further research on these associations may identify opportunities to decrease the cumulative social disadvantage that burdens people living with obesity. Additional research on clinical practice assessment of IWB, such as with WSSQ, is needed to optimize obesity management. These findings further highlight the need for comprehensive and patient‐centric management, especially for individuals with greater IWB, to enable better health outcomes. Understanding IWB in those living with obesity may lead to opportunities to identify and mitigate the negative effects of IWB on obesity disease management capability and outcomes using accessible assessment tools.

## Author Contributions

J.P., H.K., T.S., N.A., N.X. conceptualized the study. A.J. acquired and analyzed the data. S.D. and S.H. wrote the first draft of the manuscript. All authors commented on the design of the study, discussed results, provided critical revisions, and reviewed the final draft of the manuscript. All authors have read and approved the final manuscript.

## Conflicts of Interest

Scott Kahan received consultation or advisory fees from Eli Lilly and Company, Novo Nordisk, Vivus Inc., Currax Pharmaceuticals LLC, Amgen Inc., and Boehringer Ingelheim Ltd. Jamy Ard reports research support for Nestle Healthcare Nutrition, Eli Lilly, Boehringer Ingelheim, Epitomee Inc., UnitedHealth Group R&D, KVK Tech, WW, Novo Nordisk; received consulting fees from Nestle Healthcare Nutrition, Eli Lilly, Optum Labs R&D, Novo Nordisk, Intuitive, Regeneron, Brightseed, Amplifier Therapeutics, Amgen, Ingredion; member of advisory board at Novo Nordisk, Nestle Healthcare Nutrition, Eli Lilly, WW, Boehringer Ingelheim. Nadia N. Ahmad, Hong Kan, Julia P. Dunn, Tracy J. Sims, and Neena A. Xavier are employees of Eli Lilly and Company and have received stock or stock options. Soohyun Hwang, Adam Jauregui, and Sheila Drakeley are employees of Oracle Life Sciences.

## Supporting information

Supporting information
