# Supplementary material for: Exploring the Role of Internalized Weight Bias as a Metric of Cumulative Social Disadvantage Among People With Obesity or Overweight: Insights From the OBSERVE Study
Source: Obes Sci Pract. 2025 Jun 30;11(4):e70082. doi: 10.1002/osp4.70082 (PMC12207901; doi:10.1002/osp4.70082)
Supplement: Supplementary file 1 — Supporting information [file OSP4-11-e70082-s001.docx]

**Supplementary Material**

**Table S1.** Mapped survey questions to SDoH Pillars

| **SDoH Pillar** | **Characteristics** | **Survey Question** |
| --- | --- | --- |
| **Socioeconomic Position** | **Gender** | How would you describe your gender? |
|  | Male |  |
|  | Female |  |
|  | **Race/Ethnicity** | How would you describe your race? |
|  | White |  |
|  | Black |  |
|  | Asian |  |
|  | Hispanic |  |
|  | Multiracial/Other/PNA |  |
|  | **Highest education completed** | What is the highest level of education you have completed? |
|  | Some college or TS, or AA/AS degree or TS Grad. or less |  |
|  | BA/BS degree or above |  |
|  | **Household income level** | What was your total annual household income before taxes for 2021? Please include income for you personally, your spouse / partner, and any other adult in the household. Please give us your best estimate if you’re not sure. |
|  | > $75k |  |
|  | < $30k |  |
|  | $30k to ≤$75k |  |
| **Economic Stability** | **Costs of WL meds affect weight management^a^** | To what extent does each of the following prevent you from successfully keeping weight off over the long-term (at least 2 years)? |
|  | No |  |
|  | Yes |  |
|  | **Costs of lifestyle changes affect weight management^a^** |  |
|  | No |  |
|  | Yes |  |
|  | **Costs of bariatric surgery affect weight management^a^** |  |
|  | No |  |
|  | Yes |  |
| **Community and Social Interactions** | **Experiencing problems with social interactions due to weight^b^** | Please rate your level of agreement with each of the statements below about yourself: |
|  | Disagree |  |
|  | Agree |  |
|  | **Nobody to participate with for weight management journey^b^** | To what extent does each of the following prevent you from successfully keeping weight off over the long-term (at least 2 years)? |
|  | Disagree |  |
|  | Agree |  |
|  | **Negative treatment by clinician/staff due to weight^b^** | Please rate your level of agreement with each of the statements below about yourself: |
|  | Disagree |  |
|  | Agree |  |
| **Neighborhood and Physical Environment** | **Lack of access to exercise affects weight management^c^** | To what extent does each of the following prevent you from successfully keeping weight off over the long-term (at least 2 years)? |
|  | No |  |
|  | Yes |  |
| **Food Environment** | **Healthy food being too expensive affects weight management^c^** |  |
|  | No |  |
|  | Yes |  |
|  | **Limited healthy food options in neighborhood for weight management^c^** |  |
|  | No |  |
|  | Yes |  |
| **Health and System** | **Clinician discusses weight management with patient** | My healthcare provider… |
|  | Sets WL goals that feel achievable |  |
|  | Does not discuss |  |
|  | Sets WL goals that feel unachievable/ discusses weight management but doesn't set WL goals |  |
| **Other control variable** | **Prior weight management medical procedure** | Have you ever received any of the following? |
|  | No/PNA |  |
|  | Yes |  |
|  | **Baseline BMI** | BMI Calculation using: “Please enter your height.” AND Please enter your weight.” |
|  | Overweight (≥27.0 to 29.9) |  |
|  | Obesity Class I (≥30.0 to 34.9) |  |
|  | Obesity Class II (≥35.0 to 39.9) |  |
|  | Obesity Class III (≥40) |  |
|  | **Prior AOM experience** | Are you currently using, or have you ever used, any prescription weight loss medications prescribed by a healthcare provider? |
|  | Never/ |  |
|  | Don't remember |  |
|  | Have taken/Currently taking |  |
|  | **Comorbidity count** | Which of the following conditions has a doctor told you that you have or have ever had? |
|  | 0 |  |
|  | 1 |  |
|  | 2 |  |
|  | 3 |  |
|  | 4+ |  |
|  | **Depression/Anxiety** |  |
|  | Neither |  |
|  | Depression only |  |
|  | Anxiety only |  |
|  | Depression & Anxiety |  |
|  | **Age by group** | What is your age? |
|  | 40–64 |  |
|  | 18–39 |  |
|  | 65+ |  |

Note. AA/AS, associate degree; AOM, anti-obesity medications; BA/BS, bachelor's degree; BMI, body mass index; CI, confidence interval; PNA, prefer not to answer; SDoH, social determinants of health; SE, standard error; WL, weight loss; WSSQ, weight self-stigma questionnaire; TS, trade school; ‘Weight management’ refers to ‘keeping weight off over the long-term (at least 2 years)’.

^a^ "No" was collapsed from response options "Not at all," "A little bit," and "I'm not sure/Does not apply"; "Yes" was collapsed from response options "Moderately," "Quite a bit," and "Very much"; ^b^ "Disagree" was collapsed from "Strongly disagree," "Disagree," and "Neither agree nor disagree"; "Agree" was collapsed from response options "Agree" and "Strongly agree"; ^c^ "No" was collapsed from response options "Not at all" and "A little bit"; "Yes" was collapsed from response options "Moderately," "Quite a bit," and "Very much".

**Table S2.** Multivariable regression analysis for WSSQ sub-scores and SDoH covariates.

| **SDoH Pillar** | **Characteristics** | **N** | **Self-Devaluation WSSQ Score** | | | **Fear-enacted WSSQ Score** | | |
| --- | --- | --- | --- | --- | --- | --- | --- | --- |
|  |  |  | **confounder-adjusted mean differences (SE)** | **95% CI** | **p-value** | **confounder-adjusted mean differences (SE)** | **95% CI** | **p-value** |
|  |  |  | 14.67 (0.8) | 13.02, 16.31 | <0.001 | 10.90 (0.9) | 9.23, 12.58 | <0.001 |
| **Socioeconomic Position** | **Gender** | | | | |  |  |  |
|  | Male | 351 | — | — |  | — | — |  |
|  | Female | 626 | −0.17 (0.3) | −0.84, 0.51 | 0.630 | 0.27 (0.4) | −0.42, 0.95 | 0.450 |
|  | **Race/Ethnicity** | | | | |  |  |  |
|  | White | 404 | — | — |  | — | — |  |
|  | Black | 286 | −2.32 (0.4) | −3.09, −1.54 | <0.001 | −1.54 (0.4) | −2.33, −0.75 | <0.001 |
|  | Asian | 65 | −0.64 (0.7) | −1.97, 0.69 | 0.350 | 0.04 (0.7) | −1.32, 1.40 | 0.960 |
|  | Hispanic | 147 | −0.68 (0.5) | −1.63, 0.28 | 0.160 | −0.83 (0.5) | −1.81, 0.14 | 0.094 |
|  | Multiracial/Other/PNA | 75 | −1.95 (0.6) | −3.16, −0.74 | 0.002 | −1.31 (0.6) | −2.54, −0.07 | 0.038 |
|  | **Highest education completed** | | | | |  |  |  |
|  | Some college or TS, or AA/AS degree or TS Grad. or less | 676 | — | — |  | — | — |  |
|  | BA/BS degree or above | 301 | 0.43 (0.4) | −0.31, 1.17 | 0.260 | 0.70 (0.4) | −0.05, 1.46 | 0.069 |
|  | **Household income level** | | | | |  |  |  |
|  | > $75k | 230 | — | **—** |  | — | **—** |  |
|  | < $30k | 326 | 0.07 (0.5) | −0.85, 0.98 | 0.880 | 0.19 (0.5) | −0.74, 1.12 | 0.690 |
|  | $30k to ≤$75k | 421 | −0.41 (0.4) | −1.24, 0.41 | 0.320 | −0.60 (0.4) | −1.44, 0.24 | 0.160 |
| **Economic Stability** | **Costs of WL meds affect weight management^a^** | | | | |  |  |  |
|  | No | 573 | — | — |  | — | — |  |
|  | Yes | 404 | −0.14 (0.4) | −0.96, 0.69 | 0.750 | 0.16 (0.4) | −0.69, 1.00 | 0.720 |
|  | **Costs of lifestyle changes affect weight management^a^** | | | | |  |  |  |
|  | No | 381 | — | — |  | — | — |  |
|  | Yes | 596 | −0.40 (0.4) | −1.20, 0.41 | 0.340 | 0.51 (0.4) | −0.31, 1.33 | 0.220 |
|  | **Costs of bariatric surgery affect weight management^a^** | | | | |  |  |  |
|  | No | 595 | — | — |  | — | — |  |
|  | Yes | 382 | 0.76 (0.4) | −0.02, 1.54 | 0.056 | 1.00 (0.4) | 0.20, 1.79 | 0.014 |
| **Community and Social Interactions** | **Experiencing problems with social interactions due to weight^b^** | | | | |  |  |  |
|  | Disagree | 623 | — | — |  | — | — |  |
|  | Agree | 354 | 2.39 (0.4) | 1.68, 3.10 | <0.001 | 4.65 (0.4) | 3.93, 5.38 | <0.001 |
|  | **Nobody to participate with for weight management journey^b^** | | | | |  |  |  |
|  | Disagree | 508 | — | — |  | — | — |  |
|  | Agree | 469 | 1.35 (0.4) | 0.64, 2.07 | <0.001 | 1.27 (0.4) | 0.54, 2.00 | <0.001 |
|  | **Negative treatment by clinician/staff due to weight^b^** | | | | |  |  |  |
|  | Disagree | 813 | — | — |  | — | — |  |
|  | Agree | 164 | 0.55 (0.4) | −0.32, 1.42 | 0.220 | 2.37 (0.5) | 1.48, 3.26 | <0.001 |
| **Neighborhood and Physical Environment** | **Lack of access to exercise affects weight management^c^** | | | | |  |  |  |
|  | No | 546 | — | — |  | — | — |  |
|  | Yes | 431 | 0.46 (0.4) | −0.30, 1.22 | 0.230 | 1.33 (0.4) | 0.56, 2.10 | <0.001 |
| **Food Environment** | **Healthy food being too expensive affects weight management^c^** | | | | |  |  |  |
|  | No | 338 | — | — |  | — | — |  |
|  | Yes | 639 | 0.25 (0.4) | −0.52, 1.01 | 0.520 | 0.29 (0.4) | −0.49, 1.07 | 0.460 |
|  | **Limited healthy food options in neighborhood for weight management^c^** | | | | |  |  |  |
|  | No | 560 | — | — |  | — | — |  |
|  | Yes | 417 | 1.41 (0.4) | 0.66, 2.16 | <0.001 | 0.07 (0.4) | −0.70, 0.83 | 0.860 |
| **Health and System** | **Clinician discusses weight management with patient** | | | | |  |  |  |
|  | Sets WL goals that feel achievable | 226 | — | — |  | — | — |  |
|  | Does not discuss | 293 | -0.28 (0.5) | −1.16, 0.59 | 0.520 | 0.14 (0.5) | −0.76, 1.03 | 0.760 |
|  | Sets WL goals that feel unachievable/ discusses weight management but doesn't set WL goals | 458 | 0.42 (0.4) | −0.39, 1.22 | 0.310 | 0.32 (0.4) | −0.50, 1.15 | 0.440 |
| **Other control variable** | **Prior weight management medical procedure** | | | | |  |  |  |
|  | No/PNA | 895 | — | — |  | — | — |  |
|  | Yes | 82 | −1.06 (0.6) | −2.21, 0.09 | 0.070 | 0.10 (0.6) | −1.07, 1.27 | 0.860 |
|  | **Baseline BMI** | | | | |  |  |  |
|  | Overweight (≥27.0 to 29.9) | 153 | — | — |  | — | — |  |
|  | Obesity Class I (≥30.0 to 34.9) | 384 | 0.56 (0.5) | −0.36, 1.49 | 0.230 | 0.36 (0.5) | −0.58, 1.30 | 0.450 |
|  | Obesity Class II (≥35.0 to 39.9) | 217 | 0.71 (0.5) | −0.33, 1.75 | 0.180 | 0.97 (0.5) | −0.09, 2.03 | 0.072 |
|  | Obesity Class III (≥40) | 223 | 1.06 (0.5) | 0.01, 2.11 | 0.047 | 2.09 (0.6) | 1.02, 3.16 | <0.001 |
|  | **Prior AOM experience** | | | | |  |  |  |
|  | Never/  Don't remember | 779 | — | — |  | — | — |  |
|  | Have taken/Currently taking | 198 | 0.75 (0.4) | −0.06, 1.56 | 0.068 | 0.75 (0.4) | −0.07, 1.58 | 0.075 |
|  | **Comorbidity count** |  |  |  |  |  |  |  |
|  | 0 | 138 | — | — |  | — | — |  |
|  | 1 | 163 | 0.06 (0.6) | −1.08, 1.20 | 0.920 | 0.69 (0.6) | −0.48, 1.86 | 0.250 |
|  | 2 | 177 | −0.16 (0.6) | −1.37, 1.05 | 0.800 | 0.09 (0.6) | −1.14, 1.33 | 0.880 |
|  | 3 | 140 | −0.17 (0.7) | −1.46, 1.11 | 0.790 | 0.59 (0.7) | −0.72, 1.90 | 0.380 |
|  | 4+ | 359 | 0.34 (0.6) | −0.92, 1.59 | 0.600 | 0.88 (0.7) | −0.39, 2.16 | 0.170 |
|  | **Depression/Anxiety** | | | | |  |  |  |
|  | Neither | 496 | — | — |  | — | — |  |
|  | Depression only | 72 | −0.28 (0.6) | −1.55, 0.98 | 0.660 | −0.63 (0.7) | −1.92, 0.66 | 0.340 |
|  | Anxiety only | 127 | 0.75 (0.5) | −0.30, 1.79 | 0.160 | 0.87 (0.5) | −0.19, 1.94 | 0.110 |
|  | Depression & Anxiety | 282 | 0.26 (0.5) | −0.69, 1.21 | 0.590 | 1.03 (0.5) | 0.06, 2.00 | 0.037 |
|  | **Age by group** | | | | |  |  |  |
|  | 40–64 | 467 | — | — |  | — | — |  |
|  | 18–39 | 354 | 0.38 (0.4) | −0.37, 1.13 | 0.320 | 0.20 (0.4) | −0.57, 0.96 | 0.620 |
|  | 65+ | 156 | 0.06 (0.5) | −0.89, 1.02 | 0.900 | −0.67 (0.5) | −1.64, 0.30 | 0.180 |

Note. AA/AS, associate degree; AOM, anti-obesity medications; BA/BS, bachelor's degree; BMI, body mass index; CI, confidence interval; PNA, prefer not to answer; SDoH, social determinants of health; SE, standard error; WL, weight loss; WSSQ, weight self-stigma questionnaire; TS, trade school; ‘Weight management’ refers to ‘keeping weight off over the long-term (at least 2 years)’.

^a^ "No" was collapsed from response options "Not at all," "A little bit," and "I'm not sure/Does not apply"; "Yes" was collapsed from response options "Moderately," "Quite a bit," and "Very much"; ^b^ "Disagree" was collapsed from "Strongly disagree," "Disagree," and "Neither agree nor disagree"; "Agree" was collapsed from response options "Agree" and "Strongly agree"; ^c^ "No" was collapsed from response options "Not at all" and "A little bit"; "Yes" was collapsed from response options "Moderately," "Quite a bit," and "Very much".
